# A Scoping Review of the Effect of EEG Neurofeedback on Pain Complaints in Adults with Chronic Pain

**DOI:** 10.3390/jcm13102813

**Published:** 2024-05-10

**Authors:** Britt B. Schuurman, Richel L. Lousberg, Jan U. Schreiber, Therese A. M. J. van Amelsvoort, Catherine J. Vossen

**Affiliations:** 1 Department of Psychiatry & Neuro-Psychology, School for Mental Health and Neuroscience, Faculty of Health Medicine and Life Sciences, Maastricht University, 6200 MD Maastricht, The Netherlands; 2 Department of Anaesthesiology and Pain Medicine, Maastricht University Medical Centre, 6229 HX Maastricht, The Netherlands; 3 Department of Anaesthesiology, School for Mental Health and Neuroscience, Faculty of Health Medicine and Life Sciences, Maastricht University, 6200 MD Maastricht, The Netherlands

**Keywords:** EEG neurofeedback, chronic pain, pain management, scoping review

## Abstract

**Background and Aim:** Non-pharmacological treatments such as electroencephalogram (EEG) neurofeedback have become more important in multidisciplinary approaches to treat chronic pain. The aim of this scoping review is to identify the literature on the effects of EEG neurofeedback in reducing pain complaints in adult chronic-pain patients and to elaborate on the neurophysiological rationale for using specific frequency bands as targets for EEG neurofeedback. **Methods:** A pre-registered scoping review was set up and reported following the guidelines of the Preferred Reporting Items for Systematic Reviews and Meta-Analysis (PRISMA) extension for Scoping Reviews (PRISMA-ScR). The data were collected by searching for studies published between 1985 and January 2023 in PubMed, EMBASE, and PsycINFO. **Results:** Thirty-two studies on various types of chronic pain were included. The intervention was well-tolerated. Approximately half of the studies used a protocol that reinforced alpha or sensorimotor rhythms and suppressed theta or beta activity. However, the underlying neurophysiological rationale behind these specific frequency bands remains unclear. **Conclusions:** There are indications that neurofeedback in patients with chronic pain probably has short-term analgesic effects; however, the long-term effects are less clear. In order to draw more stable conclusions on the effectiveness of neurofeedback in chronic pain, additional research on the neurophysiological mechanisms of targeted frequency bands is definitely worthwhile. Several recommendations for setting up and evaluating the effect of neurofeedback protocols are suggested.

## 1. Introduction

**Figure 1:**
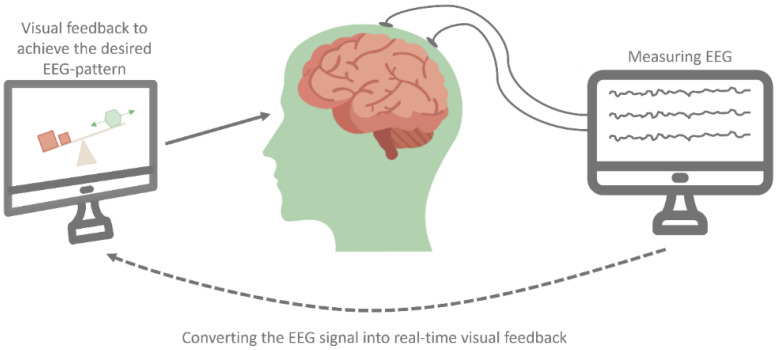
Schematic representation of EEG neurofeedback.

During the past few decades, chronic pain research has contributed to the current knowledge and led to new treatment perspectives. Nevertheless, chronic pain remains a major global health problem because of the disease burden, high prevalence, and financial costs [1,2,3]. It is generally acknowledged that chronic pain management should use a multidisciplinary approach in agreement with the biopsychosocial model [2]. In such an approach, non-pharmacological treatment options become more and more important [4]. One non-pharmacological therapy option is electroencephalography (EEG) neurofeedback, which targets brain activity. Over the last few decades, neurofeedback has been used to train individuals to control their brainwaves (see Figure 1). Throughout therapy sessions, EEG is constantly measured by placing electrodes on the scalp that record the electrical signals generated by neurons in the brain. From these raw EEG data, different frequency components can be calculated in terms of the number of waves per second (Hz). By providing subjects with real-time auditory or visual feedback regarding their brainwave activity, they can (unconsciously) learn to modify the targeted frequency band. It is thought that the modification of frequency bands can potentially lead to improvement in desired mental states, cognitive processes, and, in this case, chronic pain complaints [5,6].

In the present review, we are particularly interested in comparing the results of EEG neurofeedback in chronic-pain patients to known changes in EEG brain waves from observational (cohort) studies. Combining these results might help to recognize specific EEG neurofeedback targets for the treatment of chronic pain. After all, such a mechanism-based approach in pain treatment would be most ideal [2]. The current scoping review focused on the pain-reducing effect of EEG neurofeedback in the context of available literature with respect to EEG brain waves in chronic-pain patients.

## 2. Methods

This scoping review was preregistered on the PROSPERO database (https://www.crd.york.ac.uk/prospero/ (accessed on 21 July 2019)), with the record number CRD42019139264. It was reported following the guidelines by Preferred Reporting Items for Systematic Reviews and Meta-Analysis (PRISMA) extension for Scoping Reviews (PRISMA-ScR) [7].

### 2.1. Eligibility Criteria

Randomized controlled trials (RCTs), non-randomized controlled trials, and case-series that measured the difference in pain intensity before and after EEG neurofeedback with a validated pain questionnaire to assess the effect in chronic-pain patients were considered eligible. Adults (≥18 years) with any type of chronic pain, as identified using any recognized pain questionnaire or as diagnosed using any recognized diagnostic criterion, were included. Exclusion criteria were studies involving only healthy subjects or laboratory animals, and acute pain. Studies involving adolescents (under 18 years of age) were excluded, since the EEG can differ from adult EEG due to ongoing brain development [8]. There were no language restrictions.

### 2.2. Search Strategy

The data were collected by searching in PubMed, EMBASE, and PsycINFO, using the following most important keywords: EEG, neurofeedback, and chronic pain. The complete PubMed search strategy is presented in Appendix A, which was tailored for EMBASE and PsycINFO. Studies published between 1985 and April 2024 were searched. We subscribed to the citation alert on PubMed in order to stay informed on the latest published studies. The references of all reviewed articles were explored to include any additional studies.

### 2.3. Selection Process

The first and last authors of this study independently assessed studies for eligibility. They began by extracting data from titles and abstracts. The abstracts were included for full-text assessment when they met the criteria mentioned above. Any disagreement between the first and last authors regarding the eligibility of particular studies was resolved through a discussion with a third reviewer.

### 2.4. Risk of Bias

The risk of bias was assessed for all included studies. The Cochrane Risk of Bias tool (RoB 2.0) was used to assess RCTs based on the following 5 domains: randomization process, deviation from the intended intervention, missing outcome of the data, measurement of the outcome, and selection of the reported results [9]. For non-randomized trials, the Cochrane Risk of Bias in Non-Randomized Studies of Interventions (ROBINS-I) was used [10]. This tool evaluates 7 domains, namely bias due to confounding, bias in the selection of participants into the study, bias in classification of the intervention, bias due to deviations from the intended intervention, bias due to missing data, bias in the measurement of outcomes, and bias in the selection of reported results.

### 2.5. Synthesis of Results

Relevant data from the included studies were independently collected by the first and last author and summarized in a table, using Microsoft Office Excel 2016. The following study characteristics were manually extracted: author, year of publication, sample size, mean age, sex ratio, type of chronic pain of the study population, type of neurofeedback used, reinforced or suppressed frequency bands, location targeted by neurofeedback, number of sessions, duration per session, type of control group, pain questionnaire, significant pain reduction (yes/no), pain score pre-treatment, pain score post-treatment, pain reduction from pre- to post-treatment, pain score during follow-up, and side effects. When a study reported several outcome measures to describe multiple aspects of the pain experience, it was decided to use the outcome “pain severity”. If “pain severity” was not reported, for example, in a study population experiencing migraine [11], it was chosen to report a reduction in the frequency of the pain complaints. In accordance with the present eligibility criteria, studies with a population that partially consisted of participants aged <18 years old were also excluded. Nevertheless, in cases where the outcome measure was reported within a case series design and the study predominantly focused on an adult population, the decision was made to incorporate the study into the present review. However, it should be noted that data pertaining to individuals under the age of 18 are excluded from the present review.

## 3. Results

### 3.1. The Effects of EEG Neurofeedback on Chronic Pain Complaints

#### 3.1.1. Search Results

**Figure 2:**
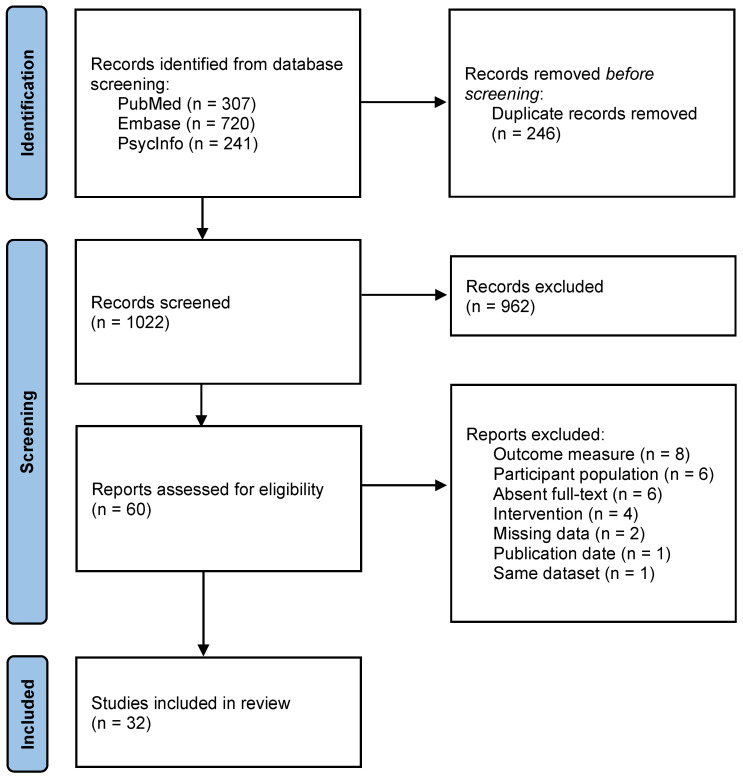
Flowchart study selection.

The initial search resulted in 1268 records, of which 1022 remained after duplicate removal (see Figure 2 for a flow diagram [12]). The evaluation of titles and abstracts led to the exclusion of 962 records that did not meet the inclusion criteria. Sixty full-text articles were assessed for eligibility. After the exclusion of another 28 articles that were not in line with the inclusion criteria, 32 studies were included in the present scoping review. Below, the characteristics of these studies are described. Then, an elaboration on the effects of the different types of neurofeedback on reducing chronic pain complaints is given. Finally, the adverse events and long-term effects are discussed.

#### 3.1.2. Characteristics of Included Studies

**Figure 3:**
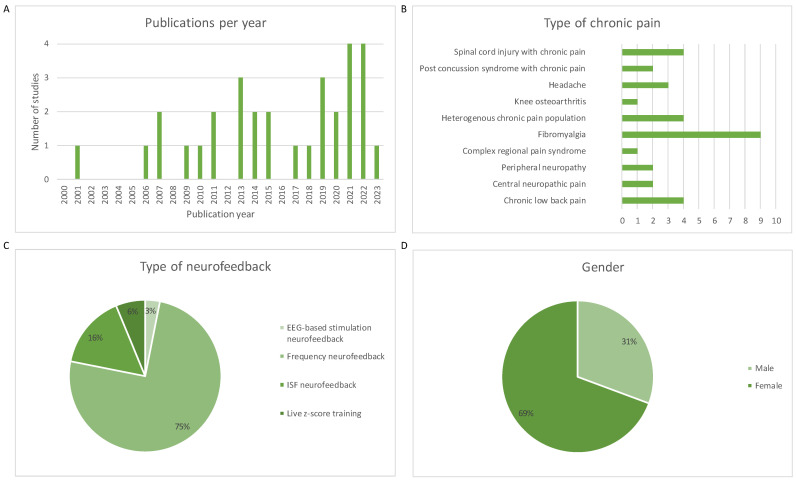
A visual representation of the study characteristics of the included studies. (**A**) The number of publications per year. (**B**) The types of chronic pain in the study population. (**C**) The frequency with which the type of neurofeedback was used as the intervention. (**D**) The gender distribution of the included studies.

All included studies were published over the past two decades. In total, the included studies investigated 1033 individuals, of which 667 were female and 295 were male. One study did not report the gender of the study population [11]. Various types of chronic pain were studied: four studies on spinal cord injury with chronic pain [13,14,15,16], two studies on chronic pain complaints with a post-concussion syndrome [17,18], three studies on headache [11,19,20], one study on knee osteoarthritis [21], four studies on heterogeneous chronic pain complaints [22,23,24,25], nine studies on fibromyalgia [26,27,28,29,30,31,32,33,34], one study on complex regional pain syndrome [35], two studies on chemotherapy-induced peripheral neuropathy [36,37], two studies on central neuropathic pain [38,39], and four studies on chronic low back pain [40,41,42,43]. In the majority of studies, frequency neurofeedback was used as the neurofeedback intervention [11,13,14,15,16,17,20,22,23,24,26,27,28,29,30,31,32,33,35,36,37,41,42]. The other studies used ISF neurofeedback (N = 5) [19,21,39,40,43], Live Z-score Training (N = 2) [18,25], and EEG-based stimulation neurofeedback (N = 1) [34]. For a complete overview of the characteristics of the included studies, see Table 1. Figure 3 was constructed to provide a visual representation of certain study characteristics.

#### 3.1.3. Risk of Bias

Three of the eleven included RCTs were evaluated as a low risk of bias, seven as a high risk, and one with some concerns (see Table 2). In Table 3, the risk of bias of non-randomized trials is assessed. One study was assessed as a low risk, twelve as a high risk, and six with some concerns. The two case reports of Orakpo and colleagues were not included in the risk-of-bias assessment [39,43].

### 3.2. Pain Reduction

#### 3.2.1. Frequency Neurofeedback

In EEG frequency neurofeedback, individuals are trained to increase or decrease specific brain oscillations. The conventional names for the EEG frequency bands, measured in hertz (Hz), are delta (<4 Hz), theta (4–8 Hz), alpha (8–13 Hz), beta (13–30 Hz), and gamma (>30 Hz). Another frequently used frequency band is the sensorimotor rhythm (SMR), ranging from 12 to 15 Hz [5,6]. All the included studies used different frequency neurofeedback protocols with respect to the reinforced or suppressed frequencies, training site, number of sessions, and duration of sessions (see Table 2, Table 3, Table 4, Table 5, Table 6, Table 7 and Table 8). An elaboration of the results of the included studies per reinforced and/or suppressed frequency band is provided below.

#### 3.2.2. Reinforce Alpha and/or SMR and Suppress Theta and/or Beta

Sixteen of the thirty included studies, comprising 475 participants, used a training protocol with a combination of upregulating alpha and/or SMR and downregulating theta and/or beta activity. Among these, 13 studies reported promising results regarding pain reduction (Table 4).

Six of these thirteen studies investigated a study population of patients with fibromyalgia [26,27,28,29,30,33]. Of these six studies, two RCTs found a statistically significant pain reduction. In the RCT by Wu and colleagues, participants reported a pain reduction from 5.16 to 3.80 in the neurofeedback group and from 4.40 to 4.24 in the control group [33]. Kayiran and colleagues observed a significantly higher pain reduction, i.e., from 8.94 to 1.64, in the neurofeedback group than in the control group (from 9.11 to 4.69) [28]. In addition, two other studies, which were non-controlled, documented a significant decrease in pain. Barbosa-Torres et al. noted a decrease from 8.4 to 6.3 [26], whereas Caro and Winter reported a reduction of 39% on a 0–10 scale [27].

Furthermore, two non-controlled studies analyzed subjects with spinal cord injury with chronic neuropathic pain, and both found a statistically significant reduction in pain in 12 out of 15 patients, and this reduction was clinically significant in 8 participants (>30% decrease) [13,16]. Another study without a control group that investigated central neuropathic pain, found a statistically significant pain reduction in five out of seven patients, which was clinically significant in four patients [38].

Two studies analyzed the effect of neurofeedback on chronic headache complaints, and both found a significant reduction in pain complaints [11,20]. More specifically, Farahani et al. compared neurofeedback, transcutaneous electrical nerve stimulation, and a control group and evaluated the effect on headache severity. They found a statistically significant difference (*p* < 0.01) between the neurofeedback and the control group. The neurofeedback group decreased in pain complaints from 5.16 to 4.18, whereas, in the control group, a slight increase (from 5.54 to 5.67) was observed [20]. In the two case series that included a heterogeneous chronic-pain population, a substantial improvement in chronic-pain complaints was reported [23,24].

Finally, three studies that did not find an effect of EEG neurofeedback on pain reduction included a heterogeneous chronic-pain population [22] or subjects with spinal cord injury with chronic pain [14,15]. In the prospective study of Birch and colleagues, no statistically significant pain reduction was observed; however, 69% of the participants did report an improvement in pain complaints [22]. 

**Table 4:** Frequency neurofeedback.

Study		Neurofeedback Protocol					Outcome					
	Authors (Year)	Reinforced and/or Suppressed Frequency Bands	Location	Number of Sessions	Duration per Session (min)	Pain Questionnaire	Significant Pain Reduction (Yes/No)	Pain Pre-Treatment (Mean (SD))	Pain Post-Treatment (Mean (SD))	Pain Reduction from Pre- to Post-Treatment (Mean (SD))	Follow-Up (Mean (SD))	Side Effects
1	Al-Taleb et al. (2019) [13]	Reinforce alpha (9–12 Hz) and suppress theta (4–8 Hz) and beta (20–30 Hz)	Between C2–C4	1–3 sessions pre-treatment and 2–105 at home	20–30	VNS (0–10)	Yes	Twelve out of fifteen participants had a statistically significant reduction in pain (*p* = 0.05). In 8 participants, this reduction was clinically significant (>30%).			Not available	Hypersensitivity in the feet and occasional headaches
2	Barbosa-Torres et al. (2021) [26]	Reinforce SMR (12–15 Hz) and suppress theta (4–8 Hz)	C4	20	15	VAS (0–10)	Yes	8.4	6.3	-	Not available	Not available
3	Birch et al. (2022) [22]	Reinforce alpha (8–13 Hz), suppress theta (4–8 Hz), beta (13–30 Hz) and high beta (20–30 Hz)	Somatosensory and prefrontal cortices	Ranging from 33 to 58 (mean 41.7)	Ranging from 23.7 to 41.6 h (mean 32.4 h)	VNS (0–10)	No	4.88	3.13	-	After 4 weeks: 3.75 (not significant)	Not available
											After 12 weeks: 3.5 (not significant)	
4	Caro and Winter (2011) [27]	Reinforce SMR (12–15 Hz) and suppress theta (4–8 Hz) and beta 22–30 Hz)	Cz	Ranging from 40 to 98 (mean 58)	Not available	Verbally reported using a 0–10 scale, where 10 was maximally abnormal	Yes	-	-	39% improvement in Global Pain compared to baseline (*p* = 0.006)	NA	Not available
5	Farahani et al. (2014) [20]	Reinforce SMR (12–15 Hz) and suppress theta (4–8 Hz) and high beta (21–30 Hz)	T3 and T4	15	30	Blanchard Headache Diary	Yes	NF 5.16 (1.69)	NF 4.18 (1.98)	-	Not available	Not available
								TENS 5.42 (1.43)	TENS 4.51 (1.35)			
								Control 5.54 (1.18)	Control 5.67 (1.18)			
6	Hassan et al. (2015) [38]	Reinforce alpha (9–12 Hz) or SMR (12–15 Hz) and suppress theta (4–8 Hz) and higher beta (20–30 Hz)	C4, C3, Cz, P4 (one electrode at the time)	20–40	40–45	VNS (0–10)	Yes	Statistically significant reduction in pain in 5 patients. Clinically significant reduction in pain in 4 patients (>30%).			After 1 month: reduced pain intensity, but increased compared to final NF session	Strong spasm of paralyzed legs
7	Ibric and Dragomirescu (2009) [23]	Reinforce beta (15–18 Hz) or SMR (12–15 Hz) and suppress theta (4–7 Hz) and high-beta (22–30 Hz)	Tailored based on localization of pain	15–145	45	VAS (0–10)	Unknown	All patients reported a substantial improvement in chronic pain.			Not available	Not available
8	Jacobs and Jensen (2015) [24]	Reinforce alpha (8–12 Hz) and slow-beta (12–15 Hz), suppress theta (4–7 Hz) and fast-beta (22–32 Hz)	Tailored based on patients’ response	22–41	30	Neurofeedback Progress Chart (0–4 Likert scale)	Unknown	All patients reported a substantial improvement in chronic pain.			Not available	Not available
9	Jensen et al. (2013) [15]	Protocol 1: reinforce alpha (8–12 Hz) and suppress theta (4–7.5 Hz).	Protocol 1: T3 and T4.	12	Not available	NRS (0–10)	No	5.95 (1.70)	5.36 (1.67)	No clinically meaningful effect (>30%)	After 3 months: 5.65 (1.90)	Not available
		Protocol 2: Reinforce 10–15 Hz and suppress beta (13–21 Hz) and theta (4–7.5 Hz).	Protocol 2: C3-A1 and C4-A2.									
		Protocol 3: Reinforce 10–15 Hz and suppress beta (13–21 Hz) and theta (4–7.5 Hz).	Protocol 3: P3-A1 and P4-A2.									
10	Jensen et al. (2013) [14]	Reinforce alpha (8–12 Hz) and suppress high-beta (18–30 Hz)	T3 and T4	1	20	NRS (0–10)	No	Neurofeedback: 4.61 (1.93)	Neurofeedback: 4.41 (2.09)	-	Not available	Not available
								Sham tDCS: 4.39 (2.07)	Sham tDCS: 4.23 (2.02)			
								tDCS: 4.19 (2.02)	tDCS: 3.92 (2.21)			
								Hypnosis: 4.27 (2.08)	Hypnosis: 3.74 (2.16)			
								Meditation: 4.44 (2.16)	Meditation: 3.96 (1.97)			
11	Kayiran et al. (2007) [29]	Reinforce SMR (12–15 Hz) and suppress theta (4–7 Hz)	C4	10	30	VAS (0–10)	Unknown	Subject 1: 8.0	Subject 1: 4.0	-	Not available	Not available
								Subject 2: 8.0	Subject 2: 3.5			
								Subject 3: 5.0	Subject 3: 2.0			
12	Kayiran et al. (2010) [28]	Reinforce SMR (12–15 Hz) and suppress theta (4–7 Hz)	C4	20	30	VAS (0–10)	Yes	Intervention: 8.94 (0.189)	Intervention: 1.64 (0.213)	-	After 8 weeks: 1.92 (0.269)	Not available
											After 16 weeks: 2.42 (0.341)	
											After 24 weeks: 2.56 (0.357)	
								Control: 9.11 (0.231)	Control: 4.69 (0.482)		After 8 weeks: 3.25 (0.269)	
											After 16 weeks: 4.47 (0.339)	
											After 24 weeks: 5.33 (0.302)	
13	Kristevski et al. (2014) [30]	Reinforce SMR (12–15 Hz) and suppress theta (4–7 Hz) and high-beta (22–30 Hz)	C4	8–16	30	VAS (0–10)	No	Statistically significant reduction in pain in 2 out of 5 patients.			Not available	Mild headache, increased pain, increased fatigue
14	Vuckovic et al. (2019) [16]	Reinforce alpha (9–12 Hz) and suppress theta (4–8 Hz) and beta (20–30 Hz)	Between C2-C4	Ranging from 3 to 48 (mean = 14)	25–30	VNS (0–10)	Yes	Twelve out of fifteen participants had a statistically significant reduction in pain. In 8 participants, this reduction was clinically significant (>30%).			Not available	Tingling sensations in toes/fingers, headaches, hypersensitivity in soles of feet
15	Walker et al. (2011) [11]	Reinforce 10 Hz and suppress 21–30 Hz	Not specified	Ranging from 12 to 32 (mean 24)	30	Headache Diary	Yes	Intervention: 54% experienced complete remission of migraines, 39% had >50% reduction in headache frequency per month.			Not available	Not available
								Control: 0% complete remission of migraines, 8% had >50% reduction in headache frequency per month.				
16	Wu et al. (2021) [33]	Reinforce alpha (8–12 Hz) and SMR (12–15 Hz) and suppress theta (4–7 Hz) and beta (18–22 Hz)	C3, C4, Cz	20	30	BPI (0–10)	Yes	Intervention: 5.16 ± 1.77	Intervention: 3.80 ± 1.80	Difference between means of change scores −2.27 to −0.52, *p* = 0.002.	Not available	Not available
								Control: 4.40 ± 2.09	Control: 4.24 ± 1.67			

#### 3.2.3. Reinforce Alpha

Four studies, with a total of 296 participants, utilized a neurofeedback training protocol to enhance alpha activity (see Table 5). Alpha activity is thought to represent a state of relaxation [5,6]. Elbogen et al. studied participants with traumatic head injury with chronic pain in a preliminary study and found a statistically significant pain reduction from 6.41 to 5.39 on a scale ranging from 0 to 10, following alpha neurofeedback [17]. More significant results regarding alpha neurofeedback were found in the pilot study of Prinsloo et al. They studied patients with chemotherapy-induced peripheral neuropathy compared to a wait-list control and observed a significant pain reduction (*p* = 0.001). The neurofeedback group reported a pre-treatment pain severity of 4.70, which decreased to 2.70 post-treatment. The waiting-list control group showed a minimal reduction in pain severity (from 4.58 to 4.25) [37]. Two other studies, however, did not report a significant pain reduction, both including chronic low back-pain patients [41,42]. More specifically, Shimizu et al. investigated different therapeutic combinations consisting of cognitive behavioral treatment, physical therapy, and neurofeedback in chronic low back-pain patients in contrast to controls in a prospective longitudinal study [42]. The subdivision of the population in early-chronic cases (diagnosed in the past year) and late-chronic cases (cases over 1 year after diagnosis) did not show a significant pain reduction as a result of neurofeedback training.

**Table 5:** Alpha neurofeedback.

Study		Neurofeedback Protocol				Outcome						
	Authors (Year)	Reinforced and/or Suppressed Frequency Bands	Location	Number of Sessions	Duration per Session (min)	Pain Questionnaire	Significant Pain Reduction (Yes/No)	Pain Pre-Treatment (Mean (SD))	Pain Post-Treatment (Mean (SD))	Pain Reduction from Pre- to Post-Treatment (Mean (SD))	Follow-Up (Mean (SD))	Side Effects
1	Elbogen et al. (2021) [17]	Reinforce alpha (not specified)	FP1	Ranging from 3 to 156 (mean 33.09)	10	Rate pain on 0–10 scale	Yes	6.41 (1.24)	5.39 (1.70)	-	Not available	Headset discomfort and drowsiness
2	Mayaud et al. (2019) [41]	Reinforce alpha-synchrony	19 electrodes	20	30	VAS (0–10)	No	4.42	Not available	-	After 6 months: 4.24	Not available
											After 12 months: 4.06	
3	Prinsloo et al. (2017) [37]	Reinforce alpha (8–12 Hz)	NA	20	45	BPI (0–10)	Yes	Intervention: 4.70 (0.27)	Intervention: 2.70 (0.38)	-	After 4 months (Prinsloo et al. (2018)): mean difference in pain severity between groups over time = 1.70 (SE 0.44) 95%CI 0.81–2.59	No negative side effects reported
								Control: 4.58 (0.27)	4.25 (0.36)			
4	Shimizu et al. (2022) [42]	Reinforce alpha (8–13 Hz)	NA	8	3–30	VAS (0–100)	No	NF: 68.9 (15.71)	NF: 65.2 (17.48)	-	Not available	Not available
								CBT: 72.2 (15.4)	CBT: 66.3 (14.21)			
								PT: 66.3 (14.21)	PT: 56.5 (14.74)			
								CBT + NF: 69.6 (14.65)	CBT + NF: 53.6 (19.73)			
								PT + NF: 72.2 (18.54)	PT + NF: 58.8 (20.89)			
								Controls: 70.7 (15.6)	Controls: 71.7 (16.3)			

#### 3.2.4. SMR Neurofeedback

SMR activity seems to occur during a relaxed, mindful state with reduced muscle tone and is recorded in the sensorimotor and pre-motor cortices [44]. This activity is created by a thalamo-cortical network and is associated with various cognitive functions [44,45]. Only one study, including 17 patients with fibromyalgia, assessed the effects of neurofeedback that merely focused on SMR (see Table 6) [32]. The participants who received active neurofeedback were divided into good responders (a mean performance level >50% of success during all sessions) and bad responders (a mean performance level <50% of success during all sessions). They concluded that all good responders achieved a significant pain reduction of 40% (*p* < 0.05). In the other two groups, a pain reduction could only be reached in two out of five bad responders and two out of eight participants from the sham group.

**Table 6:** SMR neurofeedback.

Study		Neurofeedback Protocol				Outcome						
	Authors (Year)	Reinforced and/or Suppressed Frequency Bands	Location	Number of Sessions	Duration per Session (min)	Pain Questionnaire	Significant Pain Reduction (Yes/No)	Pain Pre-Treatment (Mean (SD))	Pain Post-Treatment (Mean (SD))	Pain Reduction from Pre- to Post-Treatment (Mean (SD))	Follow-Up (Mean (SD))	Side Effects
1	Terrasa et al. (2020) [32]	Reinforce or suppress SMR (12–15 Hz)	C3, CP1, CP5	7	40	Numeric scale ranging from 0 to 100	Yes	-	-	Good-SMR responders: 39.75 ± 21.5. Significant average pain reduction > 40% in all good responders, but not in bad responders.	Not available	Not available
										Bad-SMR responders: 9.2 ± 12.6		
										Control: 16.29 ± 15.89.		

#### 3.2.5. Other Frequency Neurofeedback Protocols

Two studies used EEG frequency protocols that differed from the abovementioned categories (see Table 7). Mueller and colleagues investigated the effects of EEG-driven stimulation neurofeedback in 30 fibromyalgia patients in a non-controlled prospective study design [31]. This type of neurofeedback aims to increase the variability of the individual’s dominant frequency and to decrease the delta, theta, and alpha activity. They found a statistically significant reduction in pain intensity (*p* < 0.0001). Another group of researchers tested different neurofeedback protocols in a non-controlled study in 18 participants with complex regional pain syndrome type I [35]. The protocol rewarded a nominal bandwidth of 3 Hz and inhibited bands ranging from 2 to 13 Hz and from 14 to 30 Hz to encompass the entire spectrum. The training sites and reinforced frequencies were individualized and adjusted until the participants experienced a positive effect. All targeted training sites and frequencies were thought to be related to specific functions that eventually contribute to pain experience, such as relaxation, body awareness, obsessive thoughts, fear, etc. The authors reported a statistically significant difference (*p* = 0.001). The pain reduction was clinically relevant in half of the study population. Although the results of these two studies seem promising, both used a non-controlled design. Consequently, it is premature to state whether these neurofeedback protocols have potential for pain management.

**Table 7:** Other frequency neurofeedback protocols.

Study		Neurofeedback Protocol				Outcome						
	Authors (Year)	Reinforced and/or Suppressed Frequency Bands	Location	Number of Sessions	Duration per Session (min)	Pain Questionnaire	Significant Pain Reduction (Yes/No)	Pain Pre-Treatment (Mean (SD))	Pain Post-Treatment (Mean (SD))	Pain Reduction from Pre- to Post-Treatment (Mean (SD))	Follow-Up (Mean (SD))	Side Effects
1	Jensen et al. (2007) [35]	Reinforce a nominal 3 Hz bandwidth between 0–17 Hz and suppress 2–13 Hz and 14–30 Hz	A combination of 1–4 training sites of the following: P3-P4, FP1-FP2, T3-T4, FPO2-A2, Cz-Fz, F7-F8, F3-F4	1	30	NRS (0–10)	Yes	5.49 (2.24)	3.19 (2.72)	50% of participants had a clinically meaningful pain reduction (>30%)	Not available	Not available
2	Mueller et al. (2001) [31]	Reinforce variability of the dominant frequency and suppress delta (0–4 Hz), theta (4–8 Hz) and alpha (8–12 Hz).	Tailored based on patients’ response	Not available (minimum of 2 times a week)	1 h per session (average of 37.3 ± 15.6 h in total)	VAS (0–10)	Yes	5.4 ± 1.6	2.5 ± 1.7	-	After 3–18 months: from 6.6 ± 1.7 to 2.7 ± 1.6	NANot available

##### ILF Neurofeedback

Three studies investigated the effects of ILF neurofeedback on 89 subjects (see Table 8). ILF neurofeedback was developed by Othmer and colleagues [46,47]. This type of neurofeedback focusses on the frequencies below 0.1 Hz, without specifically reinforcing or suppressing the signal intensity. ILF oscillations seem to be related to some neurophysiological processes, such as cerebral vasomotor fluctuations, cortical excitability, and heart-rate variability [48]. The therapeutic effectiveness of ILF neurofeedback seems promising for various symptoms (for a recent review on this subject, see Bazzana 2022) [49]. In chronic pain conditions, two studies demonstrated encouraging findings. In a pilot study by Adhia et al., the effects of ILF neurofeedback in different cortical areas were investigated in chronic low back-pain patients compared to a sham group [40]. The authors report that all treatment groups showed decreasing trends in pain. In addition, the protocol which reinforced ILF oscillations in the pregenual anterior cingulate cortex (pgACC) showed a clinical meaningful pain reduction. In another study by Arina et al., ILF-neurofeedback was compared to sham neurofeedback in patients with tension-type chronic headache in a cross-over design [19]. In six out of eight patients, a clinically significant reduction in headache frequency was observed. This reduction was statistically significant in three of the six patients. Orakpo et al. published two case reports about the analgesic effects of optimizing ILF oscillations at T3-T4 and T4-P4 on chronic pain complaints. In the first case report [39], a 55-year-old woman with central neuropathic pain experienced a 40% decrease in pain complaints after 20 neurofeedback sessions. The second case report [43] described an approximately 80% decrease in pain complaints in a 31-year-old man with chronic low back pain. Finally, Mathew and colleagues analyzed the effects of simultaneously reinforcing ILF oscillations in the pgACC and suppressing ILF in the somatosensory cortex (SSC) and the dorsal anterior cingulate cortex (dACC) in patients with chronic painful knee osteoarthritis [21]. However, this pilot study reported no clinically meaningful reduction in pain severity between the chronic pain group and sham group, and no statistical analysis was performed.

**Table 8:** ILF neurofeedback.

Study		Neurofeedback Protocol				Outcome						
	Authors (Year)	Reinforced and/or Suppressed Frequency Bands	Location	Number of Sessions	Duration per Session (min)	Pain Questionnaire	Significant Pain Reduction (Yes/No)	Pain Pre-Treatment (Mean (SD))	Pain Post-Treatment (Mean (SD))	Pain Reduction from Pre- to Post-Treatment (Mean (SD))	Follow-Up (Mean (SD))	Side Effects
1	Adhia et al. (2023) [40]	Group 1: reinforce ILF	Group 1: pgACC	12	30	BPI (0–10)	Unknown	4.2 (1.8)	2.7 (1.7)	Group 1: −1.5 (95%CI: −2.3, −0.6)	After 1 week: −1.6 (95%CI: −2.2, −0.9)	Mild headaches and increased dreaming
											After 1 month: −1.9 (95%CI: −2.7, −1.0)	
		Group 2: suppress ILF	Group 2: dACC and SSC					3.3 (1.6)	2.4 (1.5)	Group 2: −0.9 (95%CI: −1.9, 0.1)	After 1 week: −0.8 (95%CI: −2.3, 0.6)	
											After 1 month: −0.8 (95%CI: −1.8, 0.2)	
		Group 3: concurrently reinforcing ILF and suppressing ILF	Group 3: pgACC and dACC + SSC					3.4 (1.3)	3.5 (1.4)	Group 3: 0.1 (95%CI: 0.4, 0.5)	After 1 week: 0.2 (95%CI: −0.6, 1.1)	
											After 1 month: −0.1 (95%CI: −0.8, 0.7)	
		Group 4: sham						3.6 (1.4)	3.3 (1.8)	Group 4: −0.3 (95%CI: −1.0, 0.3)	After 1 week: −0.8 (95%CI: −1.3, −0.3)	
											After 1 month: −1.1 (95%CI: −1.8, −0.4)	
2	Arina et al. (2022) [19]	Targets infra-low frequency EEG fluctuations (<0.1 Hz)	P4 and T4	10	Not available	McGill Pain Questionnaire	Yes	Three out of eight participants had a statistically significant reduction in headache frequency (*p* = 0.05). In 6 participants, a clinically significant reduction in the odds of having a headache occurred (>30%).			-	Not available
3	Orakpo et al. (2021) [39]	Optimize 0.15 mHz and 0.175 mHz	T3-T4 and T4-P4	20	Not available	Wong–Baker Pain Scale (0–10)	Yes	9	5	40% decrease	After 1 month: 4	Not available
											After 3 months: 2.5	
											After 1 year: 1	
4	Orakpo et al. (2022) [43]	Optimize ISF	T3-T4 and T4-P4	20	30	Wong–Baker Pain Scale (0–10)	Yes	8	1.5	>80% decrease	After 1 year: 1	Not available
5	Mathew et al. (2022) [21]	Reinforce ILF in pgACC and suppress ILF in SSC and dACC	pgACC, SSC, dACC	9	30	BPI (0–10)	Unknown	Intervention: 3.4 (SD 1.8, CI 2.2–4.6)	Intervention: 2.5 (SD 1.7, CI 1.3–3.7)	-	After 2 weeks: 2.4 (2.1)	Fatigue
								Control: 3.4 (SD 1.3, CI 2.5–4.4)	Control: 2.5 (SD 1.7, CI 1.2–3.7)		After 2 weeks: 2.6 (1.9)	

##### Live Z-Score Training

Live Z-score neurofeedback involves the ongoing comparison of the electrical activity of the brain to a normative database, resulting in real-time Z-scores. This type of neurofeedback was only used in two pilot studies (see Table 9) and enables a continuous live assessment and modulation of brainwaves without delays [50]. Koberda and colleagues described four cases with various types of chronic neuropathic pain [25]. All participants reported a substantial improvement in chronic pain. Contradictorily, a recent feasibility study of Hershaw et al. including 38 patients with a post-concussion syndrome with chronic pain did not report a significant reduction in pain intensity [18].

##### EEG-Based Stimulation Neurofeedback

Kravitz et al. investigated the effects of a combination of EEG biofeedback and subthreshold photic stimulation on fibromyalgia symptoms in 64 patients with fibromyalgia compared with a sham group (see Table 10) [34]. No significant improvement in pain complaints was reported as the result of this type of neurofeedback.

### 3.3. Long-Term Effects

Twelve of the included studies (N = 311) reported long-term effects of EEG neurofeedback on pain reduction (see Table 2, Table 3, Table 4, Table 5, Table 6, Table 7 and Table 8). Prinsloo and colleagues [36] observed that the pain reduction, as a result of neurofeedback therapy, remained significant during 4 months after the end of treatment [36]. Two other studies found that the reported pain scores slightly increased compared to the final neurofeedback session. However, this score remained lower than the baseline score [28,38]. More specifically, the study of Kayiran compared the neurofeedback group to a control group and observed that the neurofeedback group reported significantly lower pain scores than the control group during all measurement moments after 8–24 weeks post treatment [28]. Mueller et al. noted that the analgesic effect of neurofeedback remained statistically significant from 3 to 18 months post treatment [31]. Orakpo and colleagues observed a sustained analgesic effect 1 year after 20 neurofeedback sessions in two case reports [39,43]. In the study of Birch et al., the mean pain scores were reduced after 4 and 12 weeks; however, the reductions were not statistically significant [22]. Three studies showed a decreasing trend in reported pain scores, which were not statistically significant [21,40,41]. Finally, Jensen and colleagues and Hershaw et al. did not find a long-term reduction in pain complaints [15,18]. In conclusion, 6 out of 12 studies imply a favorable effect of EEG neurofeedback on long-term pain complaints. However, these 12 studies are heterogeneous with respect to study population, neurofeedback protocol and study design. The present data are insufficient to make a statement regarding the long-term effects of neurofeedback on chronic pain complaints.

### 3.4. Adverse Events

Adverse events were reported in 10 of the included studies and were primarily mild in nature [13,16,17,18,21,30,34,37,38,40]. All observed adverse events were transient and manageable. These include mild headaches, increased dreaming, fatigue, hypersensitivity in the feet or hands, increased pain, headset discomfort, drowsiness, stiffness, and muscle spasms. Notably, no severe adverse events were reported. These findings support the overall safety and tolerability of neurofeedback therapy.

## 4. Evaluating the Present Results in Context of EEG Brain Waves Associated with Chronic Pain

Most of the included studies used a neurofeedback protocol that enhanced alpha and/or SMR, while reducing theta and/or beta activity. Nine out of 16 studies using such a protocol reported a significant decrease in pain complaints. However, in light of the limited availability of controlled trials, no definitive conclusions can be made regarding the effect of reinforcing alpha and/or SMR and suppressing theta and/or beta on the reduction in the pain complaints of chronic-pain patients. Some studies evaluated the analgesic effect of reinforcing alpha activity solely. Although this effect seems promising according to the results of two preliminary studies, these results were not supported by those of two other studies. However, the quality of the evidence is poor. Five other included studies that were published more recently, focused on ILF fluctuations in their protocols. Overall, two well-designed controlled trials showed a clinically meaningful pain reduction in chronic-pain patients after ILF neurofeedback, and one could not replicate these results. Nevertheless, these promising results should be replicated in a larger study population, before conclusions on the effects of ILF neurofeedback in patients experiencing chronic pain can be drawn. The second aim of the present scoping review is to comprehend the neurophysiological rationale behind utilizing specific frequency bands as targets for EEG neurofeedback. By exploring fundamental mechanisms and processes associated with these frequencies, a deeper understanding of their potential in chronic pain management could be attained. Focusing on underlying mechanisms may facilitate the development of a mechanism-based EEG neurofeedback protocol. Below, an elaboration on the neurophysiological rationale for utilizing these specific frequency bands as a target for EEG neurofeedback, is given.

### 4.1. Alpha

Thirteen studies aimed to enhance alpha activity, based on the hypothesis that this frequency band might be associated with chronic pain. However, various systematic reviews, attempting to identify a characteristic EEG pattern of chronic pain, have reported conflicting results with respect to alpha power. Pascoal-Faria et al. found that alpha power was decreased in patients with neuropathic pain in spinal cord injury, mostly in the frontal areas [51]. Contradictory, another review by Pinheiro and colleagues observed that alpha power was increased in neuropathic-pain patients at rest [52]. A recent systematic review by Mussigman et al. discussed resting-state EEG changes including localization in patients with chronic neuropathic pain [53]. Three out of 8 studies reported an increase in the alpha power spectrum density. In three other studies, a decrease in absolute or relative alpha power was found. One of these studies suggested that increased alpha power was associated with more pain. The alpha changes were predominantly bilateral located in the fronto-centro-parietal regions. In another recent systematic review by Zebhauser et al., it was suggested that alpha power might be negatively correlated with pain intensity according to existing literature. They also found some evidence for an association between lower peak alpha frequency (PAF) and chronic pain in adults [54]. The peak alpha frequency (PAF), also referred to as the individual alpha frequency (IAF), reflects the specific frequency within the alpha band with the highest magnitude and is quantified in hertz (Hz). A higher (faster) PAF indicates a greater magnitude at the upper end of the alpha spectrum, whereas a lower (slower) PAF suggests a higher magnitude at the lower end of the spectrum. PAF does not directly assess magnitude and should not be compared with EEG alpha magnitude measures [55]. A recent study concluded that PAF in pain-free individuals can be used as a biomarker of pain sensitivity [56]. The conflicting results with respect to alpha activity might be the result of heterogeneity between studies, such as eye status (open or closed) and the use of different power measures (i.e., absolute or relative spectral power, mean alpha power, and peak alpha frequency) [53].

### 4.2. Beta and SMR

Considering the beta neurofeedback protocols, it might be plausible to subdivide the beta frequency band. Lower beta activity, sometimes called SMR power when measured in the sensorimotor cortices [5], appears to be decreased in neuropathic-pain patients [53]. In addition, our previous study suggested that low-beta activity is decreased in individuals that develop chronic pain and increased in those who recover from chronic pain [57]. It is thought that increased SMR activity is related to improved cognitive skills as a result of inhibition of somatosensory information to the cortex [58].

The higher beta activity, on the other hand, was relatively often aimed at reducing in neurofeedback protocols, which was in line with the increased power at high-beta frequencies observed in neuropathic-pain patients [53]. High beta is associated with more complicated cognitive processes, such as attentional functions, anticipation, and affective status such as anxiety and excitation [53]. Beta activity has also been referred to as the entire bandwidth (13–30 Hz). For example, Zebhauser et al. reported increased beta power in chronic-pain patients [54].

### 4.3. Theta

Theta power is usually decreased in neurofeedback studies, which seems to be in accordance with systematic reviews stating that theta power is increased in chronic-pain patients. Pinheiro et al. concluded that theta power was increased in four out of six studies in chronic-pain patients at rest [52]. This observation was supported by a review by Mussigmann et al. [53]. They found that theta power might be positively correlated to the intensity of chronic pain. In six out of ten studies, the absolute and relative theta power increased. In four of these studies, the increase in theta activity was located in the fronto-centro-temporoparietal areas. Finally, also Zebhauser confirmed this conclusion on theta activity [54]. Moreover, although non-significant, lower theta power seemed to be associated with pain relief. The found increase in theta power in chronic-pain patients has led to the development of the thalamo-cortical dysrhythmia model as an explanatory framework for chronic pain. According to this model, nociceptive inputs result in bursts of theta activity in the thalamus. In the cerebral cortex, these theta oscillations induce disinhibition of adjacent regions. Consequently, this cascade of events might induce abnormal gamma activity and ultimately chronic pain [59,60].

However, the increase in theta power in chronic-pain patients is not unanimously confirmed by all studies [59]. For example, in our recent study, we observed lower theta oscillations that were strongly related to the development of chronic pain and vice versa for individuals that recovered from chronic pain [57]. Another recent study observed that pain relief compared to baseline pain in chronic-pain patients was associated with increased theta power [61].

### 4.4. Delta and Gamma

The delta and gamma band are not frequently used in neurofeedback protocols. In two systematic reviews searching for EEG biomarkers in chronic-pain patients, no significant trends were found in delta and gamma activity [51,53]. However, these two frequency bands have been mentioned previously in chronic pain research. It was hypothesized that delta activity is decreased in individuals who develop chronic pain [57]. This hypothesis was supported by the results of a recent study that observed increased delta power associated with pain relief in chronic-pain patients [61].

With respect to gamma power, this brain wave was mentioned above as a part of the thalamo-cortical dysrhythmia model [59]. Gamma activity appears to be increased during tonic pain in pain-free participants [62,63]. In addition to a relationship between tonic pain and gamma oscillations, Zebhauser and colleagues discovered some proof for an association between higher gamma power and chronic-pain patients [54]. In addition, May and colleagues found that prefrontal gamma oscillations might have a positive association with pain intensity in chronic back-pain patients [64].

### 4.5. ILF

The focus on infralow fluctuations for neurofeedback protocols is relatively new and has not been implemented as often as brain activity ranging from 8 to 25 Hz [49]. ILF fluctuations (<0.1 Hz) of blood oxygenation levels (BOLD signals) have been found in chronic-pain patients using fMRI [65]. Given the association between ILF activity (measured with EEG) and BOLD signals (measured with fMRI), interest in ILF-oscillations has been growing [47,48]. ILF is thought to represent the interplay between functional brain networks during cognitive processes [48]. More specifically, these fluctuations play a significant role in facilitating the flexible flow of information within the brain [65]. These fluctuations were not only associated with chronic pain, but also with other neuropsychiatric disorders [65]. Although the specificity of ILF fluctuations for diseases might seem low [65], ILF neurofeedback appears to be promising for several symptoms [49]. In addition, ILF neurofeedback is not only promising in modulating brain activity, but also in influencing neurovegetative functions such as temperature regulation, heart rate and blood pressure [49]. The broad involvement of ILF fluctuations during cognitive processes, might also explain the encouraging results of ILF neurofeedback in patients with chronic pain [19,39,40,43].

### 4.6. Conclusions

In conclusion, although there seems to be consensus on enhancing alpha activity in neurofeedback protocols to treat chronic pain, the literature on the relationship between alpha activity and chronic pain remains inconclusive. A relatively limited number of studies have investigated the association between low-beta and SMR and chronic pain. Low-beta seems to be decreased in patients experiencing chronic pain, which is in line with enhancing SMR activity in neurofeedback protocols. In accordance with reducing high-beta activity in neurofeedback protocols, high-beta activity appears to be increased in chronic neuropathic pain. In addition, theta activity is usually decreased in neurofeedback, as it is found to be higher in patients with chronic pain. However, this finding regarding the theta activity is not unanimous. Delta and gamma activity are not often used in neurofeedback; however, some evidence suggests that delta activity might be decreased in chronic pain and gamma activity might be increased. Finally, the relatively new focus on ILF fluctuations seems promising in the development of chronic pain treatment, although it is not specific for chronic pain.

## 5. Discussion

The current scoping review discusses the effects of EEG neurofeedback on the reduction in chronic pain complaints in adults. Of 32 included studies, 24 studies found promising results regarding the role of EEG neurofeedback in the treatment of chronic pain. Moreover, given the mild and transient nature of adverse events, neurofeedback is considered a safe and tolerable intervention. However, because of the scarcity of well-designed controlled trials in the included data, it is too early to make definitive statements on the effects of different neurofeedback protocols in reducing chronic pain complaints on the short- and long-term. All neurofeedback protocols differed with respect to reinforced or suppressed frequencies, training site, number of sessions, and duration of the sessions. Nevertheless, the most consistent evidence was found for a neurofeedback protocol that aims to enhance alpha and SMR activity and reduce theta and high-beta activity. In addition, the effects of the relatively new ILF neurofeedback in patients with chronic pain appear to be promising, although replication of the results on larger study populations is necessary. Furthermore, a trend was observed indicating a possible long-term effect of EEG neurofeedback on pain reduction. However, more research is needed to investigate these long-term effects.

### 5.1. Interpretation and Variability of the Results

As mentioned before, the variability in results may be attributed to heterogeneity between studies, with respect to study design, characteristics of the study population, used power measures, and used protocols. In addition, other factors may complicate the interpretation of the study results. Comorbid conditions such as depression or anxiety may have a potential influence on brain activity. Since psychiatric diseases, such as depression or anxiety, are known to be associated with chronic pain [66,67,68], it seems reasonable to speculate that these factors may have a potential influence on brain activity and therefore reduce the pain experience. Interestingly, studies focusing on depression have reported that alpha activity is increased in individuals with depression [69]. Additionally, asymmetrical alpha activity has been observed, characterized by imbalances between the left and right hemisphere [69]. The presence of depressive symptoms and anxiety in individuals experiencing chronic pain might alter the neurophysiological responses associated with pain processing. Consequently, this may result in unique patterns of alpha activity that are different from those observed in individuals with chronic pain without comorbid psychiatric disorders.

### 5.2. Searching for a Neurofeedback Protocol for Chronic Pain

Based on the findings of the current scoping review, it was hypothesized that enhancing alpha activity may result in a reduction in chronic pain complaints. Furthermore, ILF fluctuations are a relatively new focus in chronic pain management and appear encouraging. However, since no clear conclusions could be drawn about the role of certain brain activity as a mechanism in the development or maintenance of chronic pain, no specific recommendations can be formulated for a neurofeedback protocol to reduce pain complaints. Further research is needed on the neurophysiological mechanisms of brain waves in chronic pain. More specifically, the role of brain activity in the chronification of pain might be a promising research field. After all, diagnosing chronic pain in a premature stage might help to maximize treatment effects [42,70].

Another recent development in neurofeedback protocols is the use of the Brain–Computer Interface (BCI) [71]. The BCI is an artificial intelligence system that recognizes patterns in EEG activity [72]. Such a system might be useful in identifying patterns in EEG activity associated with the experience of chronic pain.

The following recommendations are made in investigating the abovementioned neurofeedback protocols. First, a longitudinal study design seems valuable to analyze the role of certain brain activity in the transition to or from a chronic pain state [57]. Second, more research is needed on the complicated interaction between psychosocial variables and chronic pain and how this results in specific EEG patterns. Third, future studies should aim to standardize methodology, such as power measures and study design, to enhance reliability and comparability of the conclusions. Finally, more research is needed on delta and gamma activity and ILF fluctuations.

Eventually, as suggested before, more well-designed trials are needed to improve the quality of the present results on the analgesic effects of EEG neurofeedback [73,74,75]. For example, a cross-over, sham-controlled design, as was used in the study of Arina et al. [19], would be preferable. Alternatively, well-designed RCTs that compare EEG neurofeedback to sham neurofeedback, as was performed in four of the included studies [21,32,34,40], are advised. The guidelines of Ros and colleagues can be used to create comparable and robust experimental designs [76]. Lastly, additional investigations are necessary to study the long-term effects of EEG neurofeedback on pain reduction.

### 5.3. Strengths and Limitations

A broad overview of the effects of EEG neurofeedback on chronic pain complaints in adults is given in the present scoping review. Several reviews and meta-analyses have been previously conducted on EEG neurofeedback as a non-pharmacological treatment for chronic pain. Four reviews focused on specific chronic pain syndromes, namely fibromyalgia [77,78,79] and cancer-related pain complaints [80]. Three other recent reviews evaluated the effects of neurofeedback in heterogeneous chronic-pain populations [73,74,75]. They concluded that the available evidence in the literature was low due to multiple factors. First, the heterogeneity between studies is large with respect to neurofeedback protocols, study populations, and study designs, thus complicating the comparison between different studies. Second, most of the included studies were pilot studies, with small sample sizes.

To our knowledge, this review presents a first attempt to provide a comprehensive elaboration on the association between the analgesic effects of EEG neurofeedback and the underlying rationale for specific protocols. This endeavor might contribute to the development of a mechanism-based treatment for chronic pain. The present preregistered scoping review was reported in agreement with the recommended guideline [7]. A strength of this scoping review is the thorough search. Compared to the three existing reviews on the effects of EEG neurofeedback in patients with various types of chronic pain [73,74,75], nine other studies could be added in the present scoping review [19,21,22,26,30,31,37,40,42]. Of these nine studies, four were recent randomized, controlled studies [19,21,40,42].

Although the present findings suggest that the analgesic effects of EEG neurofeedback in chronic-pain patients are promising, it should be interpreted with care. Although a risk-of-bias assessment was performed, the lack of a thorough quality assessment is considered a limitation of the present scoping review. An important issue in researching pain management is the assessment whether the decrease in pain complaints is clinically meaningful [81]. It was attempted to describe the clinical importance of the improvement in pain complaints as a result of neurofeedback in the present scoping review. Nevertheless, describing clinical significance in an objective manner is frequently challenging. Several studies included in this review considered a pain reduction of 30% as clinically significant. Generally, a reduction of 10–20% is accepted to be “minimally important”. However, the subjective value of such a decrease is influenced by the baseline pain intensity. For example, a reduction in pain intensity from 8 (severe pain) to 6 (moderate pain) on a 0–10 Numeric Rating Scale (NRS) potentially appears to be of greater clinical significance than a decrease from 3 to 1, where both scores represent “mild pain”. Additionally, the value participants give to their pain score differs per individual [81].

Other limitations may be caused by the broad nature of the present study. For example, the generalizability of a finding in a specific group of patients to other populations or settings may be limited. The present study excluded data involving participants under 18 years of age because of the potential differences in EEG patterns due to the development of the brain compared to the EEG of adults. However, it would be interesting to study children and adolescents, since the developing brain might be more receptive to neurofeedback because of the neural plasticity and adaptability [82]. Another limitation is that some of the included studies simultaneously tested other treatments, making it more difficult to determine whether the analgesic effect is solely the result of neurofeedback treatment. In addition, Ros and colleagues described that various factors contribute to the beneficial effects of neurofeedback, such as the general advantages of cognitive training, repetition-related effects, and placebo responding [76]. Consequently, no causal relation can be drawn based on the results of the included neurofeedback studies with respect to the relation between brain waves and chronic pain.

### 5.4. Conclusions

The present scoping review focuses on the mechanisms underlying the analgesic effects of EEG neurofeedback in individuals with chronic pain. Although the results of EEG neurofeedback appear promising, the evidence is inadequate. Currently, most evidence was available for a protocol in which alpha activity is enhanced; however, the literature lacks consensus on its association with chronic pain. Additionally, other promising neurofeedback targets are SMR, theta, high-beta, and ILF. Further research on the neurophysiological mechanisms of the targeted frequency bands is worthwhile in order to draw a more definitive conclusion regarding the effectiveness of neurofeedback in chronic pain.

## Figures and Tables

**Table 1:** Characteristics of included studies.

Study		Study Design			Participants’ Characteristics			
	Authors (Year)	Intervention	Control	Randomized	Sample Size	Condition	Gender (Male/Female)	Mean Age (Years)
1	Adhia et al. (2023) [40]	ISF neurofeedback	Sham	Yes	Group 1: 15	Chronic low back pain	Group 1: 6/9	Group 1: 41.9 ± 15.8
					Group 2: 15		Group 2: 2/13	Group 2: 39.9 ± 15.4
					Group 3: 15		Group 3: 5/10	Group 3: 43.9 ± 15.4
					Group 4: 15		Group 4: 4/11	Group 4: 42.5 ± 15.4
2	Al-Taleb et al. (2019) [13]	Frequency neurofeedback	No control group	No	20	Spinal cord injury with central neuropathic pain	17/3	50.6 ± 14.1
3	Arina et al. (2022) [19]	ISF neurofeedback	Sham	Yes	8 (crossover design)	Tension type headache	1/7	30.75 ± 8.97
4	Barbosa-Torres et al. (2021) [26]	Frequency neurofeedback	No control group	No	37	Fibromyalgia	0/37	54.92 ± 7.89
5	Birch et al. (2022) [22]	Frequency neurofeedback	No control group	No	16	Heterogenous chronic-pain population	4/12	Male 52.4 and female 49.5
6	Caro and Winter (2011) [27]	Frequency neurofeedback	Treatment as usual	No	Intervention: 15	Fibromyalgia	Intervention: 1/14	Intervention: 66.7 ± 12.3
					Control: 63		Control: 13/50	Control: 50.5 ± 13.9
7	Elbogen et al. (2021) [17]	Frequency neurofeedback	No control group	No	41	Traumatic brain injury with chronic pain	35/6	38.57 ± 10.04
8	Farahani et al. (2014) [20]	Frequency neurofeedback	No treatment	Yes	Neurofeedback: 15	Primary headache	NF 8/7	NF 37.60 ± 7.462
					TENS: 15		TENS 9/6	TENS 40.73 ± 10.124
					Control: 15		Control 8/7	Control 37.33 ± 9.447
9	Hassan et al. (2015) [38]	Frequency neurofeedback	No control group	No	7	Central neuropathic pain	6/1	50 ± 4.6
10	Hershaw et al. (2020) [18]	Live z-score training	No control group	No	38	Post concussion syndrome with chronic pain	31/7	33.395 ± 8.046
11	Ibric and Dragomirescu (2009) [23]	Frequency neurofeedback	No control group	No	10	Heterogenous chronic-pain population	4/6	Ranging from 20 to 67
12	Jacobs and Jensen (2015) [34]	Frequency neurofeedback	No control group	No	3	Heterogenous chronic-pain population	1/2	Ranging from 19 to 56
13	Jensen et al. (2007) [35]	Frequency neurofeedback	No control group	No	18	Complex regional pain syndrome	2/16	40.83 (ranging from 17 to 56)
14	Jensen et al. (2013a) [15]	Frequency neurofeedback	No control group	No	10	Spinal cord injury with chronic pain	7/3	46.1 ± 12.6
15	Jensen et al. (2013b) [14]	Frequency neurofeedback	Sham tDCS	Yes	Neurofeedback: 30	Spinal cord injury with chronic pain	22/8	49.16 (ranging from 22 to 77)
					Sham tDCS: 27			
					tDCS: 28			
					Hypnosis: 29			
					Meditation: 30			
16	Kayiran et al. (2007) [29]	Frequency neurofeedback	No control group	No	3	Fibromyalgia	0/3	Ranging from 31 to 33
17	Kayiran et al. (2010) [28]	Frequency neurofeedback	Escitalopram	Yes	Intervention: 20	Fibromyalgia	0/40	Intervention: 31.78 ± 6.17
					Control: 20			Control: 32.39 ± 6.72
18	Koberda et al. (2013) [25]	LORETA and live z-score training	No control group	No	4	Various types of chronic neuropathic pain	2/2	Ranging from 46 to 59
19	Kravitz et al. (2006) [34]	EEG-based stimulation neurofeedback	Sham	Yes	Intervention: 33	Fibromyalgia	Intervention: 3/30	Intervention: 45.9 ± 9.5
					Control: 31		Control: 2/29	Control: 48.1 ± 8.9
20	Kristevski et al. (2014) [30]	Frequency neurofeedback	Wait-list control with subsequently neurofeedback	Yes	Intervention: 2	Fibromyalgia	0/5	36 ± 14.7
					Control: 3			
21	Mathew et al. (2022) [21]	ISF neurofeedback	Sham	Yes	Intervention: 11	Knee osteoarthritis	Intervention: 4/7	Intervention: 62.3 ± 8.5
					Control: 10		Control: 4/6	Control: 61.0 ± 6.7
22	Mayaud et al. (2019) [41]	Frequency neurofeedback	No control group	No	16	Chronic low back pain	0/16	37 (ranging from 15 to 52)
23	Mueller et al. (2001) [31]	Frequency neurofeedback	No control group	No	30	Fibromyalgia	3/27	50.7 ± 12.0
24	Orakpo et al. (2021) [39]	ISF neurofeedback	No control group	No	1	Central neuropathic pain	0/1	55
25	Orakpo et al. (2022) [43]	ISF neurofeedback	No control group	No	1	Chronic low back pain and sciatica	1/0	31
26	Prinsloo et al. (2017) [37]	Frequency neurofeedback	Wait-list control	Yes	Intervention: 35	Chemotherapy-induced peripheral neuropathy	Intervention: 4/31	Intervention 62 ± 9.6
					Control: 36		Control: 5/31	Control 63 ± 11
27	Prinsloo et al. (2018) [36]	Frequency neurofeedback	Wait-list control	Yes	Intervention: 35	Chemotherapy-induced peripheral neuropathy	Intervention: 4/31	Intervention 62 ± 9.6
					Control: 36		Control: 5/31	Control 63 ± 11
28	Shimizu et al. (2022) [42]	Frequency neurofeedback	Controls (not specified)	Yes	NF: 20	Chronic low back pain	NF: 12/8	NF: 61.4 ± 10.12
					CBT: 18		CBT: 10/8	CBT: 57.0 ± 12.82
					PT: 13		PT: 5/8	PT: 59.9 ± 12.72
					CBT + NF: 16		CBT + NF: 8/8	CBT + NF: 63.6 ± 9.32
					PT + NF: 10		PT + NF: 4/6	PT + NF: 57.8 ± 11.32
					Control: 20		Control: 8/12	Control: 58.9 ± 9.81
29	Terrasa et al. (2020) [32]	SMR neurofeedback	Sham	Yes	Good -SMR responders: 4	Fibromyalgia	0/17	Good -SMR responders: 54.75 ± 8.46
					Bad-SMR responders: 5			Bad-SMR responders: 53 ± 9.77
					Control: 8			Control: 56.25 ± 11.99
30	Vuckovic et al. (2019) [16]	Frequency neurofeedback	No control group	No	20	Spinal cord injury with central neuropathic pain	16/4	50.6 ± 14.1
31	Walker et al. (2011) [11]	Frequency neurofeedback	Treatment as usual	No	Intervention: 46	Migraine	NA	Ranging from 17 to 62
					Control: 25			
32	Wu et al. (2021) [33]	Frequency neurofeedback	Attention control	Yes	Intervention: 60	Fibromyalgia	Intervention: 3/57	Intervention: 48.6 ± 13.5
					Control: 20		Control: 6/14	Control: 42.2 ± 10.9

**Table 2:** Risk-of-bias assessment of the included randomized controlled trials using the RoB 2.0 tool.

	Authors (year)	Randomization Process	Deviation from Intended Interventions	Missing Outcome Data	Measurement of the Outcome	Selection of the Reported Result	Overall
1	Adhia et al. (2023) [40]	Low	Low	Low	Low	Low	Low
2	Arina et al. (2022) [19]	Low	Low	Low	Low	Low	Low
3	Farahani et al. (2014) [20]	Low	High	Low	Low	Low	High
4	Kayiran et al. (2010) [28]	Unknown	High	High	Low	Low	High
5	Kravitz et al. (2006) [34]	Some concerns	Some concerns	Low	Low	Low	Some concerns
6	Kristevski et al. (2015) [30]	Low	High	High	Low	Low	High
7	Mathew et al. (2022) [21]	Low	Low	Low	Low	Low	Low
8	Prinsloo et al. (2017) [37]	Low	High	Low	Low	Low	High
9	Prinsloo et al. (2018) [36]	Low	High	Low	Low	Low	High
10	Terrasa et al. (2020) [32]	High	High	Low	Some concerns	High	High
11	Wu et al. (2021) [33]	Low	High	High	Low	Low	High

**Table 3:** Risk-of-bias assessment of the included non-randomized controlled trials using the ROBINS-I tool.

	Authors (year)	Bias Due to Confounding	Bias in Selection of Participants into Study	Bias in Classification of Interventions	Bias Due to Deviations from Intended Interventions	Bias Due to Missing Data	Bias in Measurement Outcomes	Bias in Selection of Reported Result	Overall
1	Al-Taleb et al. (2019) [13]	High	Low	Low	Low	Low	High	High	High
2	Barbosa-Torres et al. (2021) [26]	High	Low	Low	Low	Low	High	Low	High
3	Birch et al. (2022) [22]	Some concerns	Low	Low	Low	High	Low	Low	High
4	Caro and Winter (2011) [27]	Low	Some concerns	Low	Low	Low	High	High	High
5	Elbogen et al. (2021) [17]	Low	Low	Some concerns	Low	Low	Low	Low	Some concerns
6	Hassan et al. (2015) [38]	Some concerns	Low	Low	Low	Low	Low	Low	Some concerns
7	Hershaw et al. (2020) [18]	Low	Some concerns	Low	Low	Some concerns	Low	Low	Some concerns
8	Ibric and Dragomirescu (2009) [23]	High	High	Low	Some concerns	Some concerns	Low	Some concerns	High
9	Jacobs and Jensen (2015) [24]	High	Low	Some concerns	Some concerns	Low	Low	Low	High
10	Jensen et al. (2007) [35]	High	Some concerns	Some concerns	Low	Low	Low	Low	High
11	Jensen et al. (2013) [15]	Low	Some concerns	Low	Low	Low	Low	Low	Some concerns
12	Jensen et al. (2013) [14]	Low	Low	Low	Low	Low	Low	High	High
13	Kayiran et al. (2007) [29]	Low	Low	Low	Low	Low	Low	Low	Low
14	Koberda et al. (2013) [25]	High	High	Low	Low	Low	High	High	High
15	Mayaud et al. (2019) [41]	Low	Some concerns	Some concerns	Low	Low	Low	Low	Some concerns
16	Mueller et al. (2001) [31]	High	Some concerns	High	Low	Low	High	Some concerns	High
17	Shimizu et al. (2022) [42]	Low	Low	Low	Some concerns	Low	High	Low	High
18	Vuckovic et al. (2019) [16]	Some concerns	Some concerns	Low	Low	Some concerns	Low	Low	Some concerns
19	Walker et al. (2011) [11]	High	High	High	Low	Low	High	High	High

**Table 9:** Live Z-score neurofeedback.

Study		Neurofeedback Protocol				Outcome						
	Authors (Year)	Reinforced and/or Suppressed Frequency Bands	Location	Number of Sessions	Duration per Session (min)	Pain Questionnaire	Significant Pain Reduction (Yes/No)	Pain Pre-Treatment (Mean (SD))	Pain Post-Treatment (Mean (SD))	Pain Reduction from Pre- to Post-Treatment (Mean (SD))	Follow-Up (Mean (SD))	Side Effects
1	Hershaw et al. (2020) [18]	Not available	19 electrodes	5–20	10–30	Chronic Pain Grade Questionnaire	No	48.696 (14.728)	45.650 (13.686)	-	After 11–15 weeks: 46.670 (14.873)	Mild headache and fatigue
2	Koberda et al. (2013) [25]	Not available	19 electrodes	10–65	30	Unknown	Unknown	-	-	All patients reported a substantial improvement of chronic pain complaints.	Not available	Not available

**Table 10:** Stimulation based neurofeedback.

Study		Neurofeedback Protocol				Outcome						
	Authors (Year)	Reinforced and/or Suppressed Frequency Bands	Location	Number of Sessions	Duration per Session (min)	Pain Questionnaire	Significant Pain Reduction (Yes/No)	Pain Pre-Treatment (Mean (SD))	Pain Post-Treatment (Mean (SD))	Pain Reduction from Pre- to Post-Treatment (Mean (SD))	Follow-Up (Mean (SD))	Side Effects
1	Kravitz et al. (2006) [34]	Not available	21 electrodes	22	Not available	Fibromyalgia Impact Questionnaire (0–9)	No	Intervention: 6.27 (2.41)	Intervention: 5.23 (2.34)	-	Not available	Fatigue, pain, drowsiness, stiffness, muscle spasm
								Control: 6.43 (1.79)	Control: 5.57 (2.23)			

## Data Availability

The original contributions presented in the study are included in the article; further inquiries can be directed to the corresponding author.

## Appendix A

Search PubMed

((“Neurofeedback”[Mesh] OR Neurofeedback*[tiab] OR alpha feedback*[tiab] OR EEG feedback*[tiab] OR Brainwave feedback*[tiab] OR Electroencephalography biofeedback*[tiab] OR Electroencephalography feedback*[tiab] OR brainwave biofeedback*[tiab] OR EEG biofeedback*[tiab] OR EEG bio feedback*[tiab] OR Alpha biofeedback*[tiab] OR Alpha bio feedback*[tiab] OR neurobiofeedback*[tiab])) AND (“Chronic Pain”[Mesh] OR chronic Pain*[tiab] OR “Pain”[Mesh] OR pain*[tiab] OR “Pain Management”[Mesh] OR pain management*[tiab] OR ache*[tiab] OR physical suffering*[tiab] OR fibromyalgia[tiab] OR “Fibromyalgia”[Mesh] OR musculoskeletal disease*[tiab] OR phantom limb*[tiab] OR arthritis[tiab] OR Spondyloarthritis[tiab] OR spondylarthritis[tiab] OR spondylitis[tiab] OR osteoarthritis[tiab] OR rheumatic[tiab] OR rheumatism[tiab] OR arthropathy*[tiab] OR osteoarthrosis[tiab] OR arthrosis[tiab] OR “Peripheral Nervous System Diseases”[Mesh] OR peripheral nervous system Disease*[tiab] OR neuropath*[tiab] OR PNS disease*[tiab] OR peripheral nerve disease*[tiab] OR peripheral nervous disease*[tiab] OR peripheral nervous system Disorder*[tiab] OR PNS disorder*[tiab] OR peripheral nerve disorder*[tiab] OR peripheral nervous disorder*[tiab] OR neuritis[tiab] OR polyneuritis[tiab] OR mononeuropath*[tiab] OR neuralg*[tiab] OR nerve compression*[tiab] OR nerve entrapment*[tiab] OR nervous entrapment*[tiab] OR nerve constriction*[tiab] OR nerve injur*[tiab] OR polyneuritis[tiab] OR radiculoneuritis[tiab] OR polyradiculoneuritis[tiab] OR nerve fiber inflammation*[tiab] OR polyneuropath*[tiab] OR radiculopath*[tiab] OR ischialgia[tiab] OR Sciatica[tiab] OR “Headache Disorders”[Mesh] OR cephalic syndrome*[tiab] OR headache*[tiab] OR migraine*[tiab] OR cephalgia*[tiab] OR cephalea*[tiab] OR cephalalgia*[tiab] OR “Colonic Diseases, Functional”[Mesh] OR Crohn*[tiab] OR colitis[tiab] OR colon disease*[tiab] OR colon diverticulosis[tiab] OR colon fistula*[tiab] OR colorectal disease*[tiab] OR irritable colon*[tiab] OR functional colonic disease*[tiab] OR colonic disease*[tiab] OR irritable bowel syndrome*[tiab] OR irritable colon syndrome*[tiab] OR IBD[tiab] OR inflammatory bowel disease*[tiab] OR “Endometriosis”[Mesh] OR endometriosis[tiab] OR endometrioses[tiab] OR endometrioma[tiab] OR myalgia[tiab] OR fibrositis[tiab] OR contracture*[tiab] OR chondropath*[tiab] OR enthesopath*[tiab]).
